# Long-term outcomes after endovascular aortic treatment in patients with thoracic aortic diseases

**DOI:** 10.1590/1677-5449.202201562

**Published:** 2023-11-20

**Authors:** Antonio Carlos Brandi, Carlos Alberto dos Santos, Josélia Menin Brandi, Marcio Antonio dos Santos, Paulo Henrique Husseine Botelho

**Affiliations:** 1 Faculdade de Medicina de São José do Rio Preto – FAMERP, São José do Rio Preto, SP, Brasil.; 2 Faculdade de Medicina de Marília – FAMEMA, Marília, SP, Brasil.

**Keywords:** aneurysm, aortic arch, dissection, thoracic endovascular aortic repair, aneurisma, arco aórtico, dissecção, correção endovascular da aorta torácica

## Abstract

**Background:**

Endovascular treatments for thoracic aortic diseases have been adopted rapidly, and long-term studies are relevant for durability evaluation.

**Objective:**

To evaluate the long-term results of a prospective observational study of endovascular treatment in patients with thoracic aortic diseases who underwent percutaneous implantation of self-expandable endoprostheses.

**Methods:**

Procedural success was defined as the absence of endoleak into the aneurysm or dissection-induced false lumen, no migration, and no conversion to open surgery. Intraoperative, postoperative, and late postoperative outcomes were evaluated in terms of complications, mortality, and evolution of the endoprosthesis over a follow-up of up to 179 months (median: 46 months).

**Results:**

A total of 150 endoprostheses were implanted in 112 patients. Primary success was observed in 100 (82.14%) patients. Immediate mortality occurred in 7 patients (6.25%). Late mortality occurred in 31 patients (27.68%), 10 (8.93%) of whom died from cardiovascular causes, 12 (10.71%) from non-cardiovascular causes, and 2 (1.78%) from natural causes, while 7 (6.25%) had no diagnosis for cause of death. Types I, II, and IV endoleaks occurred during hospitalization in 4 (3.57%), 5 (4.46%), and 3 (2.68%) patients, respectively. Late types I and IV endoleaks occurred in 5 (4.46%) and 3 (2.68%) patients respectively. Twenty-two patients (19.64%) had clinical complications in the immediate postoperative period. Actuarial survival free from death from cardiovascular causes was 79.3% (95% confidence interval, 67.0-91.7%) at 132 months.

**Conclusions:**

The low levels of intraoperative and postoperative complications demonstrate that endovascular treatment is safe and effective. The high rate of late survival for these critically ill patients indicates that the endovascular technique is beneficial for treatment of thoracic aortic diseases in terms of long-term outcomes.

## Resumo

**Contexto:**

Os tratamentos endovasculares para doenças da aorta torácica têm sido adotados rapidamente e estudos de longo prazo são relevantes para avaliação de durabilidade.

**Objetivo:**

Avaliar através de estudo observacional e prospectivo, os resultados a longo prazo do tratamento endovascular em pacientes com doenças da aorta torácica submetidos a implante percutâneo de endoprótese autoexpansível.

**Métodos:**

O sucesso do procedimento foi definido como ausência de vazamento interno para o aneurisma ou falso lúmen induzido por dissecção, ausência de migração e sem necessidade de conversão para cirurgia aberta. Os resultados intraoperatórios, pós-operatórios e tardios foram avaliados quanto a complicações, mortalidade e evolução da endoprótese em um seguimento de até 179 meses (mediana de 46 meses).

**Resultados:**

Um total de 150 endopróteses foram implantadas em 112 pacientes. Sucesso primário foi observado em 100 (82,14%) pacientes. Mortalidade imediata ocorreu em sete pacientes (6,25%). A mortalidade tardia ocorreu em 31 pacientes (27,68%), dos quais 10 (8,93%) morreram por causas cardiovasculares; 12 (10,71%), por causas não cardiovasculares; dois (1,78%), por causas naturais e sete (6,25%) não tiveram a causa da morte diagnosticada. Vazamentos tipo I, II e IV ocorreram durante a internação em quatro (3,57%), cinco (4,46%) e três (2,68%) pacientes, respectivamente. Vazamentos tardios tipo I e IV ocorreram em cinco (4,46%) e três (2,68%) pacientes, respectivamente. Complicações clínicas no pós-operatório imediato foram observadas em 19,64% dos pacientes. A sobrevida atuarial por causas cardiovasculares foi de 79,3% em 132 meses.

**Conclusões:**

Os baixos índices de complicações intra e pós-operatórias demonstram que o tratamento endovascular é seguro e eficaz. A alta taxa de sobrevida em 132 meses em pacientes críticos indica que a técnica é benéfica para o tratamento de doenças da aorta torácica em resultados a longo prazo.

## INTRODUCTION

Thoracic aortic aneurysm and aortic dissection are the most frequent diseases of the aorta and may require surgical treatment.^1,2^ Other, less common conditions affecting the thoracic aorta and that may also require surgical treatment include penetrating ulcers, intramural hematomas, pseudoaneurysms, coarctations, and traumatic lacerations with or without aortobronchial or aortoesophageal fistulae.^3^

Diseases of the thoracic aorta may be managed via clinical or surgical treatment, depending on the type of disease and the aortic segment affected. In cases with complications such as aortic rupture, poor perfusion of organs, or persistent pain even with pain medication, the indication is open surgical repair with cardiopulmonary bypass. However, despite advances in surgical techniques, materials, anesthetic management, and postoperative care, morbidity and mortality rates in patients with thoracic aortic diseases remain high.^4^ Over the past three decades, endovascular techniques have become a successful therapeutic strategy to improve the results of surgical treatment in patients with aortic diseases and may offer therapeutic alternatives to open surgery for patients without clinical conditions.^5-7^

The introduction of the technique of endovascular repair of the thoracic aorta by Dake et al.,^7^ in 1994, marked the beginning of a new era in the treatment of various conditions that affect this critical aortic segment. Since then, the technique has been successfully applied in many countries for the treatment of aneurysms^8-10^ and dissections of the thoracic aorta.^8,10,11^

In many countries around the world, the number of endovascular procedures performed to treat aortic diseases already surpasses that of conventional open surgery procedures.^12^ In Brazil, endovascular treatment of thoracic aortic diseases is currently performed at several different centers. However, there are few reports regarding the medium and long-term outcomes of these procedures.^11^ Thus, the present study aimed to assess the long-term outcomes of endovascular treatment in patients with thoracic aortic diseases who underwent implantation of self-expandable stent-graft endoprostheses.

## MATERIALS AND METHODS

### Study design

This study employed a prospective observational study model, following the STROBE (Strengthening the Reporting of Observational Studies in Epidemiology) rules for clinical research, available at: https://www.strobe-statement.org/.

The Cardiac Surgery team at São José do Rio Preto Hospital de Base performed patient selection. Patients were diagnosed based on the results of computed tomography (CT). One or more additional tests were performed to confirm these diagnoses, such as magnetic resonance imaging (MRI), transthoracic echocardiography, transesophageal echocardiography, and aortography.

**Figure 1:**
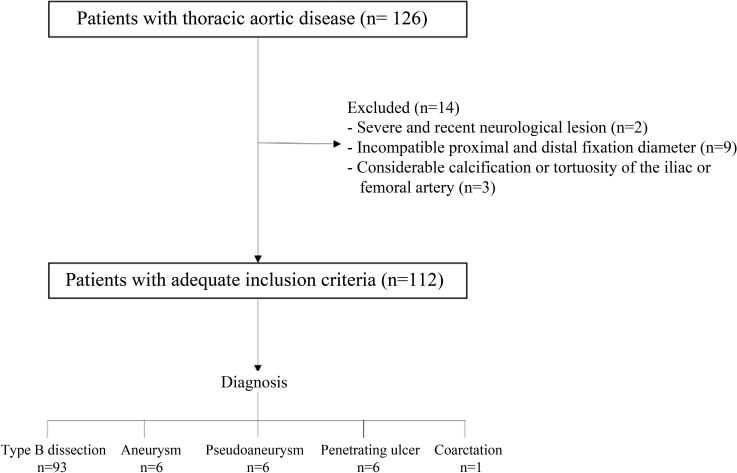
Study flow-chart.

From October 1998 to August 2013, 126 patients with thoracic aorta diseases were recruited. Fourteen (11.1%) of them were excluded because they were candidates for surgical repair, as illustrated in Figure 1. A total of 112 patients met the anatomical inclusion criteria for this procedure as follows: aortic aneurysms with a diameter > 6 cm or with aortic dilation > 5 cm in patients with Marfan syndrome, aortic neck diameter of 18-45 mm, absence of circumferential thrombus; dissections, intramural hematomas, or penetrating ulcers associated with intractable pain, progression of the dissection, signs of impending rupture, or evidence of organ ischemia; acute traumatic aortic lesions with possible rupture; and aortic coarctation. In addition, the proximal landing zone must be within Ishimaru zones 0-4.^13^

### Endoprosthesis characteristics

Initially, stainless steel endoprostheses were used (Braile Biomédica, São José do Rio Preto, Brazil), with diameters of 24-45 mm (median, 33 mm) and lengths of 70-130 mm (median, 90 mm). We later switched to polyester-coated Nitinol self-expandable endoprostheses (Braile Biomédica) when these became available, with diameters of 22-46 mm (median 35) and lengths of 40-230 mm (median, 115 mm).

Endoprostheses were chosen based on measurements obtained from the diagnostic MRI and CT images of the aorta and confirmed in the hemodynamics room at the time of the procedure. The diameter of the endoprosthesis to be implanted was always 20% larger than the diameter of the proximal site of endoprosthesis fixation in the aorta.

### Technical procedures

The patients underwent surgery under general anesthesia or local anesthesia with intravenous sedation, depending on their clinical condition and disease severity. Non-invasive blood pressure monitoring was performed in the left arm, and systolic pressure was maintained at around 80 mmHg. Subsequently, cefazolin was administered for antibiotic prophylaxis. During the procedure, anticoagulation was induced with heparin (1 mg/kg), followed by neutralization with protamine.

Angiographic control for positioning the endoprosthesis was achieved using a pigtail catheter inserted into the aorta via the right radial or right brachial artery. The access routes used to release the endoprostheses were the right and left common femoral arteries, the right and left external iliac arteries, and the right common carotid artery.

The procedure was deemed successful or primarily successful when no endoleaks into the aneurysm or dissection-induced false lumens were observed. Additionally, the procedure was considered successful when no migration of the endoprosthesis was observed, and no conversion to open surgery was necessary.

The treatment for the different types of endoleaks observed followed the guidelines set forth by the Brazilian Society of Cardiovascular Surgery, the American Heart Association, the European Association for Cardiothoracic Surgery, the European Society of Cardiology, and the European Association of Percutaneous Cardiovascular Interventions.^14-16^ Positioning of the endoprosthesis in the thoracic aorta was defined according to the Ishimaru classification.^13^

### Surgical strategy

The surgical techniques of extra-anatomic bypass of supra-aortic vessels and occlusion of the left subclavian were used to treat aortic diseases affecting the aortic arch or those initiating very close to the emergence of the left subclavian artery, respectively, concurrently with or prior to implantation of the endoprosthesis, enabling better positioning.

Furthermore, it was necessary to perform additional surgical procedures such as angioplasty of coronary arteries concurrently with endoprosthesis implantation in patients presenting serious obstructions in these arteries. Additional endoprosthesis placement was required for patients with severe arterial disease in the infra-renal abdominal aorta.

With the exception of cases with coarctation of the aorta, the same standardization was maintained for the extent of coverage of the aorta. The stump of the aorta proximal to the diseased segment should have at least 2 cm for anchoring the endoprosthesis. On the other hand, when this length was insufficient, extra-anatomical bypass of the supra-aortic vessels was chosen prior to the endoprosthesis.

### Patient follow-up

Long-term complications, progression of the underlying disease, and survival were evaluated during postoperative follow-up. Follow-up visits were scheduled for 30 days, 3 months, and every 6 months thereafter for up to 11 years postoperatively. During the follow-up visits, patients underwent examinations by CT, MRI, and aortography, alone or in combination. All data were collected for subsequent assessment.

### Statistical analysis

Data were described in terms of descriptive statistics including absolute frequency, percentage, mean, standard deviation, and median. Percentage survival over the course of the follow-up (up to 132 months) was evaluated in terms of the actuarial Kaplan-Meier curve. Logistic regression analysis was performed to determine potential clinical predictors. The treatment effect was measured in terms of odds ratio (OR) or relative risk (RR) with a 95% confidence interval (CI). An alpha error of 5% was admitted (*P* ≤ 0.05) for a variable to be considered a risk factor, and the model was rebuilt after correcting for all non-risk factors.

## RESULTS

Since the aim of our study was to assess the long-term results of self-expandable endoprosthesis implantation, all results are shown regardless of the underlying disease. The mean duration of the implantation procedure was 72.66 ± 43.36 minutes (range, 30-240 minutes), while the mean postoperative stay in the intensive care unit was 28.07 ± 32.12 hours (range, 0-96 hours).

A total of 150 endoprostheses were implanted in 112 patients over the course of the study period. A total of 61 (40.66%) of these endoprostheses were made of stainless steel, while 89 (59.33%) were made of nitinol. The number of endoprostheses implanted per patient ranged from 1 to 4 (median, 1). A total of 90 (80.36%) patients received one endoprosthesis each, while 18 (16.07%) patients received two endoprostheses each, 3 (2.68%) patients received three endoprostheses each, and 1 (0.89%) patient received four endoprostheses. The lengths of the endoprostheses implanted ranged from 40 to 230 mm (median, 110 mm), and their diameters ranged from 24 to 46 mm (median, 34 mm).

Of the 112 patients assessed, 93 (83.03%) were diagnosed with type B aortic dissection (BAD), 6 (5.36%) with trauma-related aortic disease, 6 (5.36%) with aneurysm, 6 (5.36%) with pseudoaneurysm, 6 (5.36%) with penetrating ulcers, and 1 with coarctation (0.89%). A total of 88 (78.57%) patients were male, and 24 (21.43%) were female. In this study population, age ranged from 24 to 83 years, with a mean of 58.75 ± 12.81 years and a median of 60 years (Table 1).

**Table 1:** Demographic and Clinical Characteristics of Patients Who Underwent Implantation of Aortic Endoprostheses (n = 112).

**Variable**	**Value**
**Sex**	
Male	88 (78.57%)
Female	24 (21.43%)
**Age, years**	
Average	58.75
Standard deviation	12.81
**Diagnosis**	
Type B dissection	93 (83.03%)
Aneurysm	6 (5.36%)
Pseudoaneurysm	6 (5.36%)
Penetrating ulcer	6 (5.36%)
Coarctation	1 (0.89%)
**Comorbidity**	
Hypertension	95 (84.82%)
Diabetes mellitus	21 (18.75%)
Coronary artery disease	19 (16.96%)
Chronic renal failure	14 (12.50%)
Marfan syndrome	1 (0.89%)
Smoking	54 (48.21%)

Data are given as total count (percentage) unless otherwise specified. n: number of individuals.

The following comorbidities were noted in our study population: systemic arterial hypertension in 95 patients (84.82%); chronic renal failure (CRF) in 14 patients (12.50%); coronary artery disease in 19 patients (16.96%); DM in 21 patients (18.75%); and smoking in 54 patients (48.21%) (Table 1).

**Figure 2:**
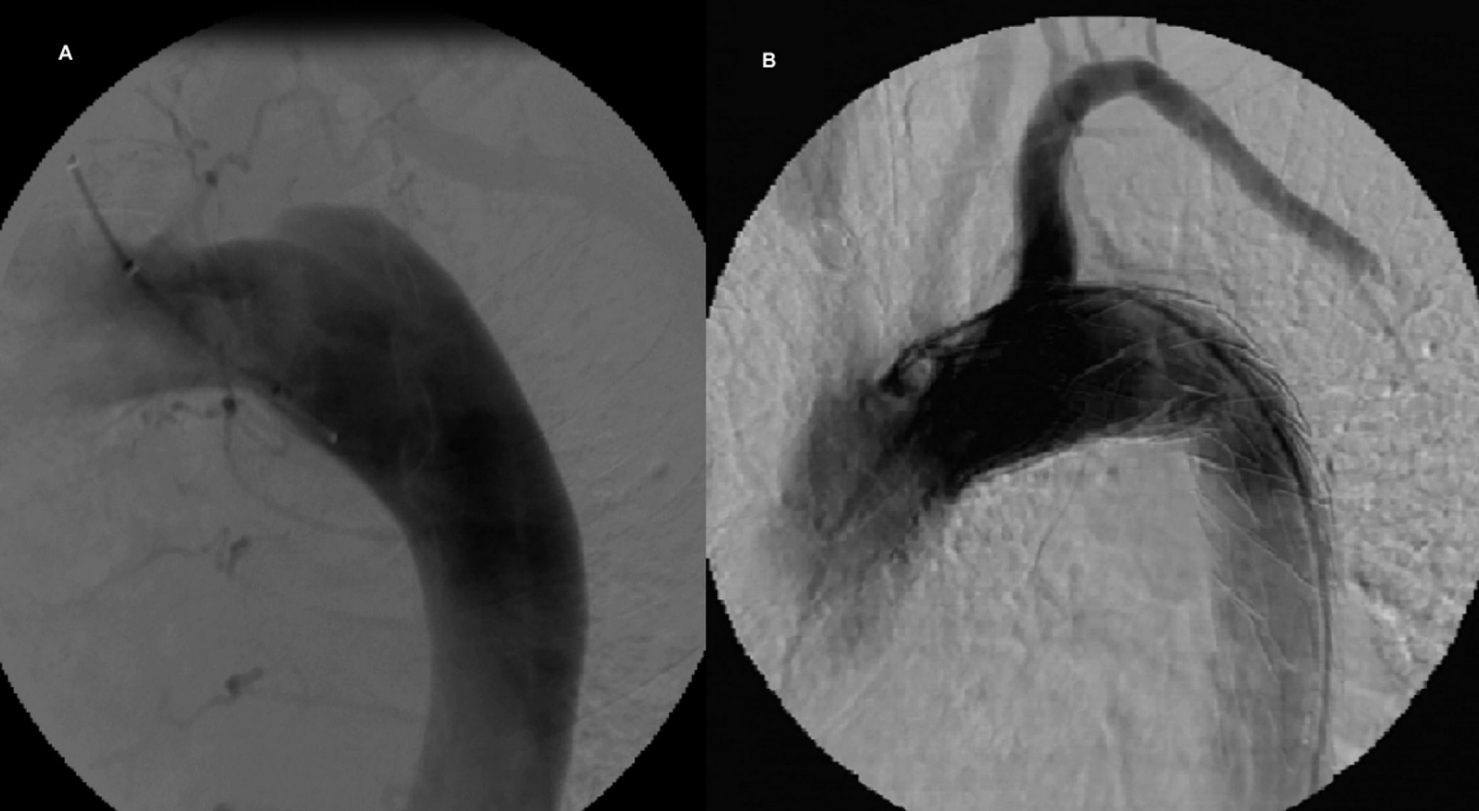
Angiography of the Thoracic Aorta before and after Endoprosthesis Implantation in a Patient with Aortic Dissection. (A) Descending aortic dissection; (B) Exclusion of the dissection-induced false lumen by the endoprosthesis.

**Figure 3:**
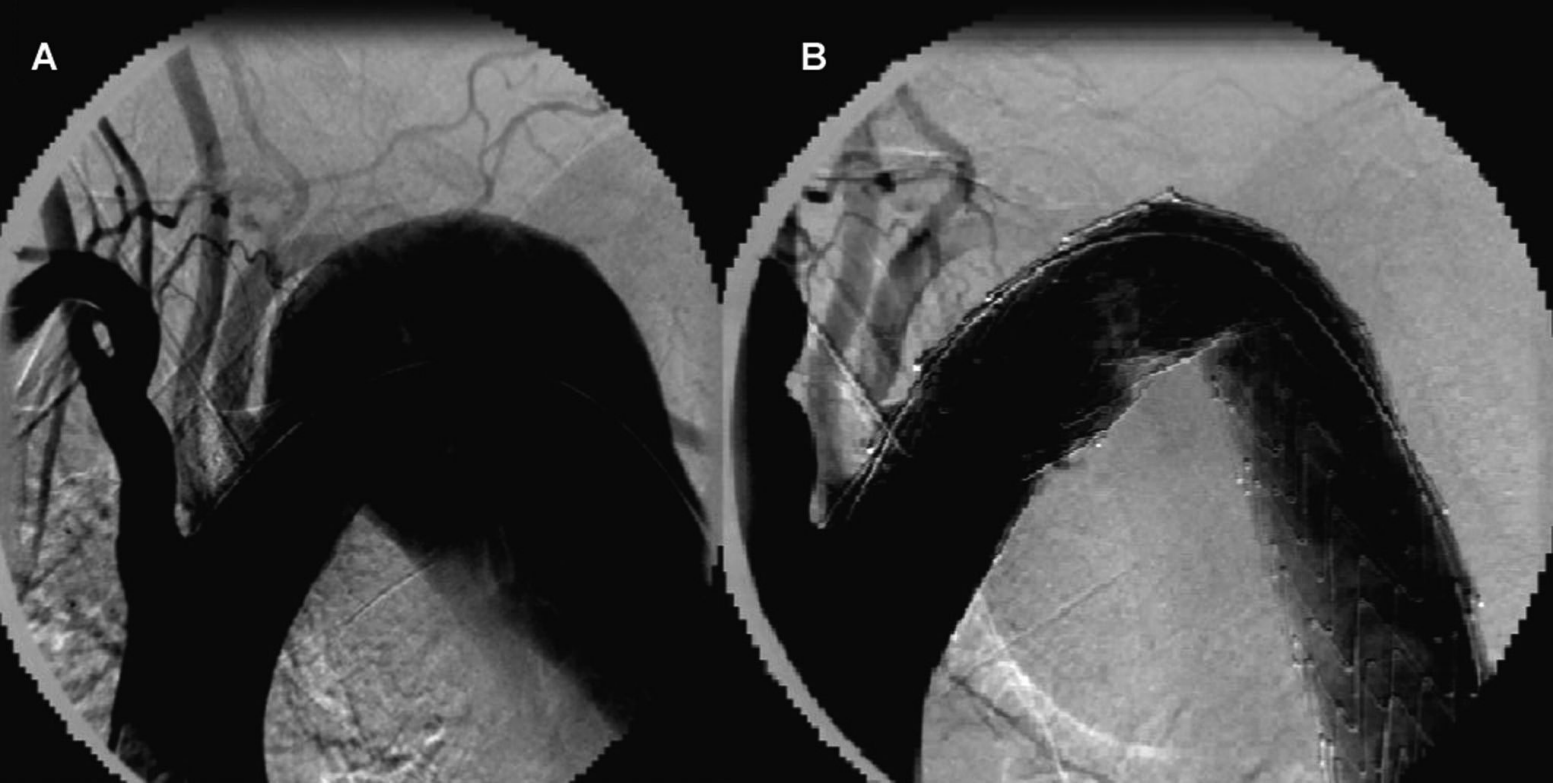
Angiography of the Thoracic Aorta before and after Endoprosthesis Implantation in a Patient with Aneurysm. (A) Large aneurysm in the descending portion; (B) Complete exclusion of the aneurysm after placement of the endoprosthesis.

**Figure 4:**
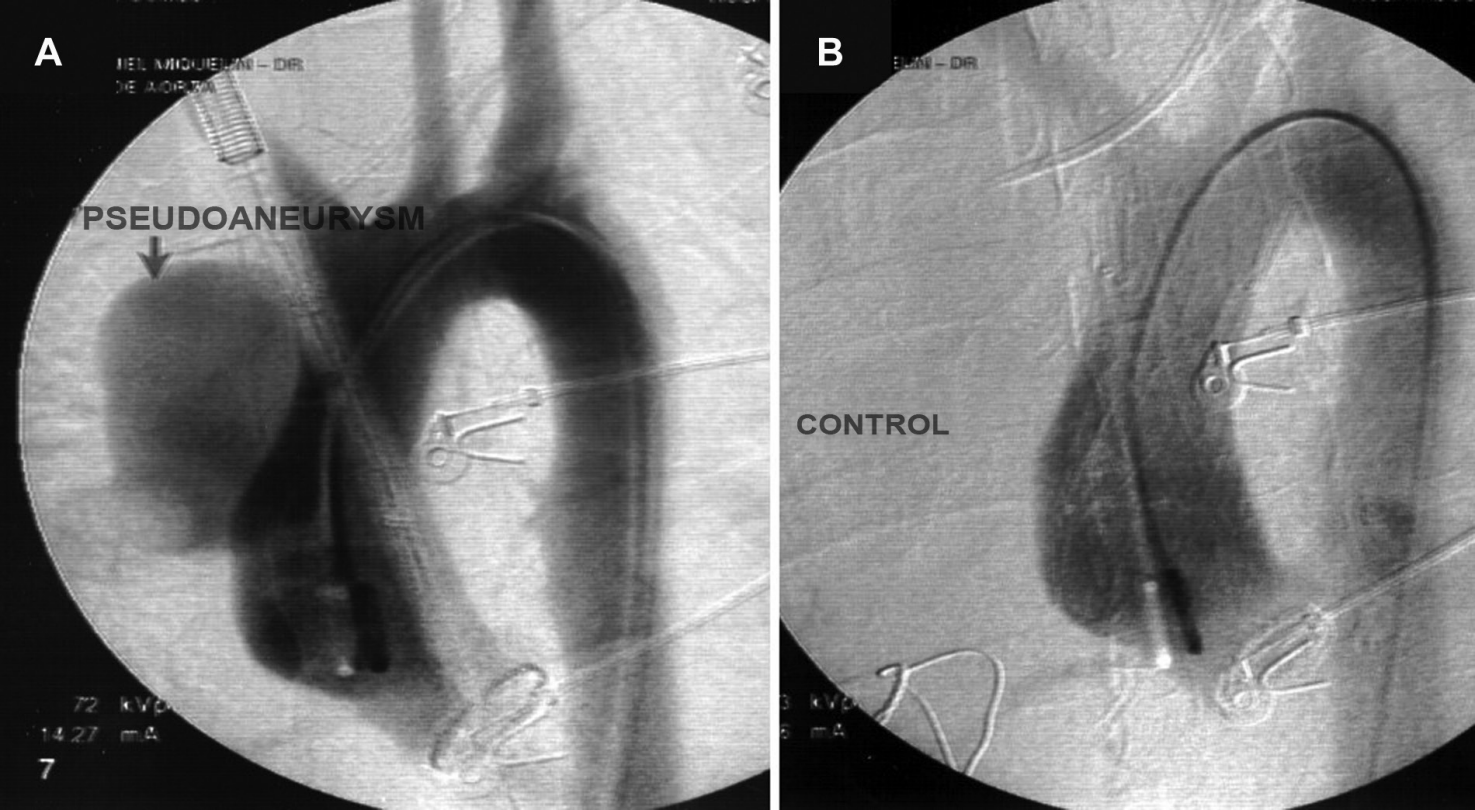
Angiography of the Thoracic Aorta before and after Endoprosthesis Implantation in a Patient with Pseudoaneurysm. (A) Pseudoaneurysm in the ascending aorta; (B) Exclusion of the pseudoaneurysm by the endoprosthesis.

**Figure 5:**
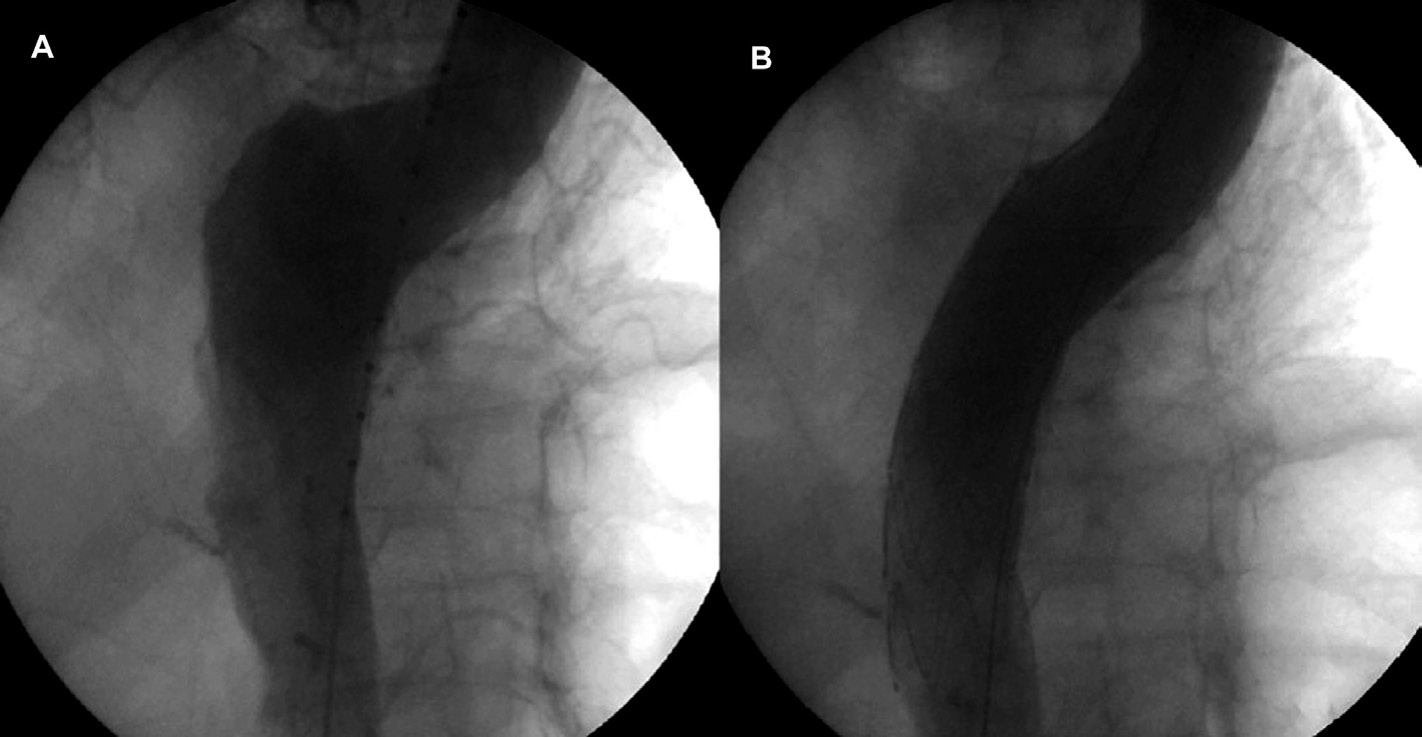
Angiography of the Descending Thoracic Aorta before and after Endoprosthesis Implantation in a Patient with Penetrating Ulcer. (A) Penetrating ulcer; (B) Occlusion of the penetrating ulcer by the endoprosthesis.

The endovascular procedure was performed with primary success, achieving the release of the endoprosthesis in its intended place and with adequate angiographic results, in 100 (89.28%) of the 112 patients who underwent implantation. The following outcomes were achieved: occlusion of the false lumen in the dissection (Figure 2); complete exclusion of the aneurysm (Figure 3); complete exclusion of the dilation of the pseudoaneurysm (Figure 4); occlusion of the ulcer (Figure 5); and repair of coarctation.

According to the Ishimaru classification, the implanted endoprostheses were positioned as follows: 7 (6.25%) endoprostheses were positioned in Zone 0; 14 (12.50%) in Zone 1; 34 (30.35%) in Zone 2; 41 (36.60%) in Zone 3; and 16 (14.28%) in Zone 4.

In-hospital mortality occurred in 7 patients (6.25%). Cardiovascular causes including hemorrhagic stroke, aortic-esophageal fistula, aortic rupture, and ischemic stroke were responsible for the death of 5 of these patients (4.46%). Non-cardiovascular causes including pneumonia and multiple trauma were responsible for the death of the other two patients (1.78%).

Among the in-hospital complications (Table 2), type I endoleak occurred in 4 patients (3.57%), type II in 5 patients (3.57%), and type IV in 3 patients (2.68%). Of these, 3 patients with type II endoleak and 3 patients with type IV endoleak showed complete resolution on radiographs at 12 months after implantation.

**Table 2:** Postoperative Complications within 30 Days of Implantation of Self-expandable Endoprostheses for Treating Thoracic Aortic Diseases (n = 112).

**Complications**	**Value**	**%**
**Endoleak**	**12**	**10.71**
Type I	4	3.57
Type II	5	4.46
Type IV	3	2.68
**Aortic dissection**	**1**	**0.89**
**Neurological changes**	**4**	**3.57**
Ischemic stroke	1	0.89
Hemorrhagic stroke	1	0.89
Right-sided temporary hemiparesis	1	0.89
Temporary hemiparesis of the lower right limb	1	0.89
**Pulmonary complications**	**9**	**8.03**
Pneumonia	8	7.14
Pulmonary embolism	1	0.89
**Renal failure**	**3**	**2.68**
Acute renal failure	3	2.68
**Complications related to surgical access**	**3**	**2.68**
Surgical wound infection	2	1.79
Laceration of the common femoral artery	1	0.89
**Post-implantation syndrome**	**2**	**2.68**

n: number of individuals.

Another complication observed during hospitalization was retrograde dissection of the ascending aorta, diagnosed in a patient who underwent conventional open surgery on the fifth postoperative day (Table 2).

A total of 22 patients (19.64%) had clinical complications in the immediate postoperative period. Specifically, 9 (8.03%) patients had pulmonary complications, of whom 8 (7.14%) had pneumonia and 1 (0.89%) had pulmonary embolism. Furthermore, 4 (3.57%) patients had manifestations of neurological disorders, including one (0.89%) with an ischemic stroke, one (0.89%) with a hemorrhagic stroke, one (0.89%) with temporary right-sided hemiparesis, and one (0.89%) with temporary hemiparesis of the right lower limb. Three (2.67%) patients developed acute renal failure. Two patients presented infection of the surgical incision and two patients developed post-implantation syndrome. Finally, a laceration of the arterial access occurred in one patient (Table 2).

Among the late complications, type I endoleaks were observed in 5 patients (4.46%), appearing within 2, 3, 18, 42, and 70 months postoperatively. Three patients (2.67%) had type II endoleaks, which appeared within 27, 30, and 36 months postoperatively.

Eight of the patients who developed type I endoleak at any point during hospitalization or follow-up underwent repeat endoprosthesis implantation. One of them was reoperated on the ninth postoperative day and the other seven were treated later. One patient with type I endoleak refused surgery and died after 55 months of an unknown cause.

Eleven (12.64%) of the 87 patients with BAD had endoleaks while hospitalized, of whom 4 (36.36%) had type I endoleaks, 5 (45.45%) had type II, and 3 (27.27%) had type IV endoleaks. A total of 8 such patients presented with late endoleak, 5 of whom (62.50%) had type I leaks and 3 of whom (37.50%) had type II leaks. Only 1 (16.66%) of the 6 patients with aneurysms had a late type I endoleak. Only 1 (16.66%) of the 6 patients who had pseudoaneurysm had a type IV endoleak while hospitalized. The remaining 13 patients (6 with aortic ulcer, 6 with trauma-related aortic disease, and 1 with coarctation) did not have endoleaks (Supplementary Material, Table S1).

The following results were obtained for the relationship between the incidences of endoleak types I, II, and IV and the proximal endoprosthesis fixation area. Two (22.22%) of the 9 patients with type I endoleaks had the endoprosthesis positioned in Zone 1, 3 (33.33%) in Zone 2, another 3 (33.33%) in Zone 3, and 1 (11.11%) in Zone 4. Three (37.5%) of the 8 patients with type II endoleaks had the endoprosthesis positioned in Zone 1, 2 (25%) in Zone 2, another 2 (25%) in Zone 3, and 1 (12.5%) in Zone 4. One (33.33%) of the 3 patients who had type IV endoleaks had the endoprosthesis positioned in zone 2, and 2 (66.66%) had it in zone 3.

A total of 32 patients (42.1%) had occlusion of the left subclavian artery due to the endoprosthesis, and none had symptoms related to ischemia in the left arm. Six extra-anatomic supra-aortic vessel bypass procedures were performed for the subsequent placement of the endoprosthesis in the aortic arch, with no neurological changes resulting from the procedures. With respect to previous aortic procedures, 8 (10.5%) patients had previously received endoprostheses in the thoracic aorta, while 12 (15.8%) had undergone prior surgery on the ascending aorta.

Late mortality occurred in 31 (27.68%) patients, of whom, 10 (8.93%) died of cardiovascular causes, 12 (10.71%) of non-cardiovascular causes, 2 (1.78%) of natural causes, and 7 (6.25%) of undiagnosed causes (Supplementary Material, Table S2).

**Figure 6:**
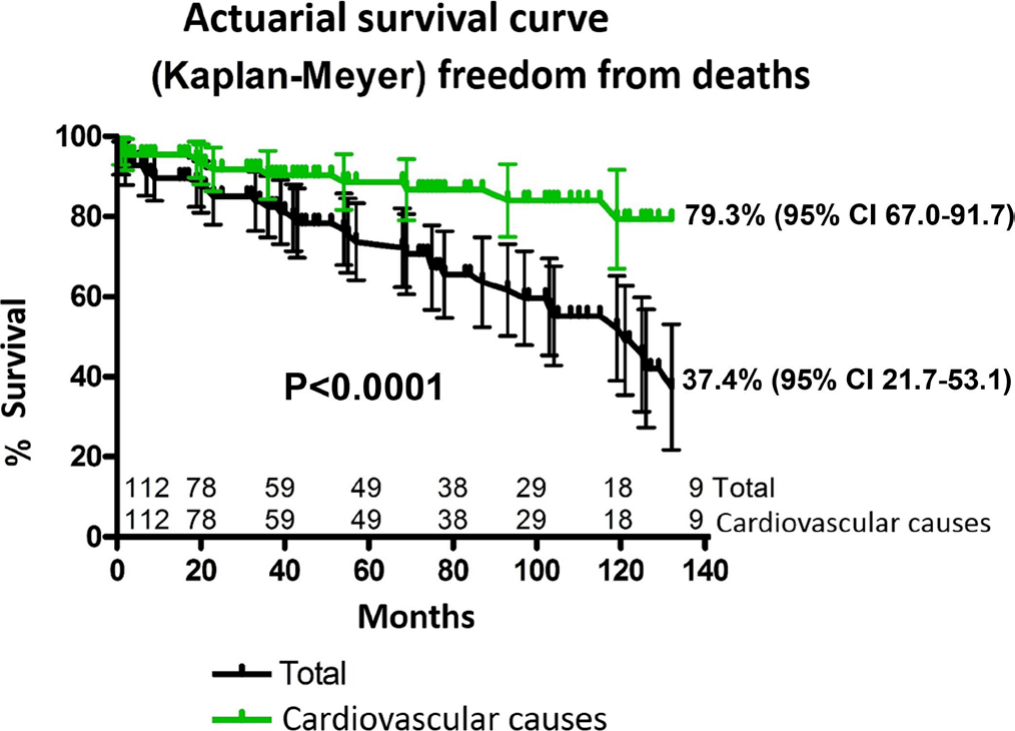
Actuarial Survival Curve after Implantation of Self-expandable Endoprosthesis for 112 Patients with Thoracic Aortic Diseases.

Follow-up duration ranged from 1 to 179 months, with a median of 46 months. Actuarial survival was 79% (95% CI, 67.0% to 91.7%) at 132 months. Of the 21% of patients who died, 37.4% (95% CI, 21.7% to 53.1%) died of cardiovascular causes (Figure 6).

The logistic regression analysis showed that chronic renal failure (CRF) was the only risk factor that was associated with a significantly higher risk of mortality (Table 3).

**Table 3:** Logistic Regression Identifying Potential Risk Factors for Mortality in Patients who Underwent Implantation of Self-expandable Endoprostheses (n = 112).

**Risk factors**	***P*-value**	***P*-value**	**RR (95% CI)**
Systemic arterial hypertension	0.4172		
Smoking status	0.9982		
Coronary artery disease	0.2083		
Diabetes mellitus	0.0502		
Chronic renal failure	0.0006*	0.0004*	3.175 (2.156-4.674)
Endoleak	0.9943		

RR: relative risk; CI: confidence interval; n: number of individuals;

* Statistically significant.

## DISCUSSION

After the past 3 decades of established use, endovascular techniques have shown exciting results in the treatment of thoracic aortic diseases, and the scope of their indications has greatly expanded.^3,17^ The short and medium-term outcomes suggest that endovascular procedures provide an advantage over conventional open surgery for the treatment of diseases of the thoracic aorta,^4^ which is partly related to the fact that cardiopulmonary bypass and aortic clamping are avoided. In addition, thoracotomy, blood transfusions, prolonged surgery time, and manipulation of the lungs greatly increase the morbidity and mortality associated with conventional surgery, especially in high-risk surgical patients, who account for the majority of patients indicated for this type of surgery.^4,14,16^

In the present study, approximately 83.03% of our population had BAD, versus a maximum of 50%, which is the proportion of patients with degenerative aortic aneurysms included in previous studies.^17,18^ One possible explanation for the large number of dissections compared to aneurysms is that the endovascular service at the Hospital de Base was one of the first to be accredited by the SUS. Therefore, there were several emergency referrals from different regions of the country. Eight of these patients had already undergone some kind of surgery to correct the ascending aorta and the aortic arch, which hindered navigation of the guidewire and displacement of the tip of the endoprosthesis release device in the ascending aorta. Nevertheless, we noted no complications.

In this series of patients, the primary endoprosthesis implantation success rate was 89.28% (100 of 112), in line with multicenter studies suggesting rates between 81.5% and 100%. Nevertheless, if we consider only type I and type III endoleaks, the primary success rate reaches 96.4%, which is in line with certain other reports.^9,15^ Approximately 40% of our patients with BAD had partial thrombosis of the false lumen at 12 months after implantation of the endoprosthesis, particularly for cases with dissections that extended into the abdominal aorta. These data are consistent with the literature that shows they occur in up to half of patients and are because of the greater angulation of the aorta in this segment, irregularities of its walls, and persistence of retrograde flow from the left subclavian artery, even after its closure with the endoprosthesis.^8-10,19^

The overall in-hospital mortality was 6.25% (7 patients), which is similar to previously reported mortality rates in such patients (5%-8.4%).^1,3,9^ Cardiovascular causes accounted for the majority of deaths (5 patients). It is of note that the underlying disease in 4 of the 5 patients who died of cardiovascular causes was BAD.

In-hospital endoleaks were found in 12 (10.71%) patients, while late endoleaks occurred in 8 (7.14%) patients, in agreement with previous reports regarding the incidence of endoleaks (ranging from 0 to 44%).^3,9,18^ Radiological monitoring of these patients showed that there was spontaneous thrombosis of the false lumen within 12 months of implantation in 3 of the patients with type II endoleak and in all 3 patients with type IV endoleak. In our series, the main cause of type II endoleak was retrograde flow from the left subclavian artery when occluded by the endograft. However, this type of leak resolved following thrombosis of the proximal portion of that artery.

Stroke occurred in two (1.78%) cases, one of which was ischemic (0.89%) and was probably caused by manipulating the guidewire and endoprosthesis in the aorta. However, this incidence rate is comparable with that reported in another study (1.7%) and lower than that reported for conventional open surgery (approximately 9%).^14,17^

Logistic regression analysis of mortality data showed that CRF was the only risk factor that showed a significantly higher risk of mortality. Preoperative CRF was present in 14 of our patients, 12 of whom died during follow-up. Although statistically significant, only 3 patients had mortality of cardiovascular origin. This high mortality in patients with CRF can be explained by the large number of comorbidities that occur concurrently with the disease, such as diabetes, high blood pressure (hypertension), cardiomyopathy, and cardiovascular and cerebrovascular diseases, in addition to serious complications related to dialysis. In large studies, CRF also appears as one of the main risk factors for mortality in aortic endovascular procedures.^8^

At the end of 132 months, the survival rate related to cardiovascular events was 79.3%, whereas in the previous reports, it was 85.01% at 112 months.^20^ In this study, the overall mortality was around 63% after 132 months, which is in line with previous studies in the literature, noting that the vast majority of these patients had type B aortic dissection with large peculiar anatomical variations, re-entry orifices, and aortic remodeling. In a series of 300 patients undergoing the endovascular procedure, Wiedemann et al.^9^ found that the mortality rate after such a procedure was 44% at 120 months and that the percentage was 38% for open surgery.

Our results regarding long-term outcomes demonstrate the safety and significant advantage of percutaneous endovascular procedures for the treatment of thoracic aortic diseases. Nowadays, endoprostheses have improved with customization for each patient and each specific disease and they can be branched in order to enable management of the aortic arch and visceral vessels.

## LIMITATIONS

This study lost participants over the 132 months of follow-up. However, the number of final participants nevertheless constitutes a considerable sample size that supports the results of the statistical analysis. This study is also an aggregated analysis of several disease conditions, such as aortic aneurysms, dissections, pseudoaneurysms, penetrating ulcers, and coarctation. Nevertheless, it analyzes a dataset from a large series of endovascular treatments in patients with thoracic aortic diseases and it was appropriate to include these patients together to make it possible to extract results and conclusions from this heterogeneous patient population. In addition, the results reflect outcomes from a real institutional setting, showing the evolution of cases over 132 months of follow-up.

## CONCLUSION

In the context of the latest technological advancements and increasing reports that demonstrate the safety and efficacy of endovascular procedures for treating thoracic aortic diseases, the indications for endovascular treatment have been consolidated. The low levels of intraoperative and postoperative complications demonstrate the efficacy and safety of endovascular treatment. Furthermore, the good survival rate observed for these critically ill patients, even after 132 months of follow-up, suggests that endovascular treatment can greatly benefit patients with thoracic aortic diseases.

## Supplementary Material

Supplementary material accompanies this paper.

Table S1. Aortic diseases versus endoleak.

Table S2. Causes of late mortality in 31 of 112 patients who underwent implantation of self-expanding endoprostheses for treatment of thoracic aortic diseases.

This material is available as part of the online article from https://doi.org/10.1590/1677-5449.202201562
