# Supplementary material for: Long-term outcomes after endovascular aortic treatment in patients with thoracic aortic diseases
Source: J Vasc Bras. 2023 Nov 20;22:e20220156. doi: 10.1590/1677-5449.202201562 (PMC10706006; doi:10.1590/1677-5449.202201562)
Supplement: Table S1. [file jvb-22-e20220156-Suppl.pdf]

Supplementary Material

Table S1. Aortic diseases versus endoleak.

Disease	Total	Hospital endoleak	Late endoleak
Type B dissection	87	11 (12.64%)	8 (9.19%)
		Type I 4 (36.36%)	Type I 5 (62.5%)
		Type II 5 (45.45%)	Type II 3 (37.5%)
		Type IV 3 (27.27%)	
Aneurysm	6		Type I 1 (16.66%)
Pseudoaneurysm	6	Type IV 1 (16.66%)	
Aortic ulcer	6		
Trauma	6		
Coarctation	1		

Table S2. Causes of late mortality in 31 of 112 patients who underwent implantation of self-expanding endoprostheses for treatment of thoracic aortic diseases.

Cause of intervention	Late Mortality	Patient No.	%
	**Cardiovascular Causes**	**10**	**8.93**
Type B dissection	AMI	3	2.68
Descending aortic ulcer	hemorrhagic stroke	1	0.89
Descending aortic ulcer	Stroke	1	0.89
Type B dissection	Rupture	1	0.89
Type B dissection	Type A dissection	1	0.89
Type B dissection	Stroke	1	0.89
Descending aortic ulcer with fistula			
Descending aortic ulcer with aortobronchial fistula	Rupture	1	0.89
Descending aortic aneurysm	CHF	1	0.89
	**Non-cardiovascular causes**	**21**	**18.79**
Type B aortic dissection	CRF + Pneumonia	1	0.89
Type B aortic dissection	Pneumonia	1	0.89
Type B aortic dissection	Kidney transplant	1	0.89
Type B aortic dissection	Gastric cancer	1	0.89
Type B aortic dissection	COPD + Sepsis	1	0.89
Type B aortic dissection	CRF	2	1.78
Type B aortic dissection	Alcoholism	1	0.89
Type B aortic dissection	Rectal cancer	1	0.89
Type B aortic dissection	Cholecystitis + Sepsis	1	0.89
Descending aortic ulcer	Perforated peptic ulcer	1	0.89
Descending aortic aneurysm	Pneumonia	1	0.89
Type B aortic dissection	Natural	2	1.78
Type B aortic dissection	Without diagnosis	7	6.25

AMI = acute myocardial infarction; CHF = congestive heart failure; COPD = chronic obstructive pulmonary disease; CRF = chronic renal failure.
